# miR-339-3p regulated acute pancreatitis induced by caerulein through targeting TNF receptor-associated factor 3 in AR42J cells

**DOI:** 10.1515/biol-2020-0084

**Published:** 2020-12-19

**Authors:** Qi Wang, Shaofeng Liu, Zhen Han

**Affiliations:** Department of Gastroenterology, The Yijishan Hospital of Wannan Medical College, Room 505, Unit 3, Building 1, Yiyuan Community, No. 109, Tuanjie West Rd, 241001, Wuhu, Anhui, China

**Keywords:** acute pancreatitis, miR-339-3p, TRAF3, caerulein, apoptosis

## Abstract

Acute pancreatitis (AP) is an inflammatory disease with high morbidity and mortality. The regulation mechanism of miRNA is involved in the production and development of various diseases, but the regulation mechanism of miRNA in AP is still not fully elucidated. The expression of miR-339-3p was detected using quantitative real-time PCR. The levels of TNF-α, IL-1β, and IL-6 were detected using enzyme-linked immunosorbent assay. Cell apoptosis was measured using flow cytometry. The protein expressions of TNF receptor-associated factor 3 (TRAF3), Bcl-2, C-caspase 3, Bax, p-p38, and p38 were measured using western blot. Luciferase reporter assay and RNA immunoprecipitation assay were applied to ensure that miR-399-3p targeted TRAF3. Caerulein promoted the expression of TNF-α, IL-1β, and IL-6, enhanced the expression of C-caspase 3 and Bax while inhibited Bcl-2 protein expression. Meanwhile, caerulein also reduced the expression of miR-339-3p and induced the expression of TRAF3 in rat pancreatic acinar cells. miR-399-3p transfection inhibited the levels of TNF-α, IL-1β, and IL-6 and C-caspase 3 and Bax protein expression as well as suppressed cell apoptosis, while increased Bcl-2 protein expression in caerulein-induced AP. TRAF3 has been verified as a target of miR-339-3p. Interestingly, the reduction of miR-399-3p inhibited the p38 pathway, which was impaired by the upregulation of TRAF3. In addition, the suppression effects of miR-339-3p on cell inflammation and apoptosis in caerulein-induced AP were reversed by enhancing TRAF3 expression. In this study, *in vitro* model of AP was characterized by strong inflammation and cell apoptosis. We have first demonstrated the regulatory network of miR-339-3p and TRAF3. Overexpression of miR-339-3p inhibited cell inflammation and cell apoptosis in caerulein-induced AP through modulating TRAF3 expression via the p38 pathway, providing a new therapeutic target in the treatment of AP.

## 1 Introduction

Acute pancreatitis (AP) is a sudden pancreatic inflammatory response accompanied by strong pain, mainly caused by necrosis of pancreatic acinar cells. AP is still fatal despite intensive treatment [1,2,3]. With the development of gene sequencing and biotechnology, the research on the mechanism of AP has also been greatly developed. It also provides an important theoretical basis for improving treatment methods and developing molecular therapy. However, the pathogenesis of AP has not yet been fully explained.

miRNA-related regulatory networks and mechanisms have been reported to play a crucial role in the mechanisms of diseases [4]. miRNA, as a non-coding small RNA, plays a major role in mRNA degradation or translational inhibition by binding to the mRNA 3′-UTR [5]. Various studies have shown that miRNA, as an important regulator, is involved in cellular processes in a variety of diseases, including GC, breast cancer, HCC, NCSLC, and pancreatitis [6,7,8,9]. In AP, different miRNAs have different expression patterns. In example, the high expression of miR-21 in AP promoted apoptotic activity, and the promotion of miR-22 and miR-135a may induce the apoptosis of pancreatic acinar cells by repressing ErbB3 and Ptk2 expression [10,11]. Growing evidence determined that miRNAs took part in cell progression widely, including cell proliferation, migration, inflammatory response, apoptosis and necrosis, and autophagy [12,13,14].

A variety of miRNA regulatory networks interact to jointly regulate the occurrence, immune response and inflammation of AP [15,16,17,18]. In addition, miR-216a, miR-7, miR-9, miR-122, and miR-141 could act as potential markers of AP [19,20]. Interestingly, Wu et al. reported that miR-339-3p decreased cell inflammation and cell apoptosis in mice with AP through Anxa3 by activation of the Akt/mTOR signaling pathway [21]. However, the regulatory mechanism of miR-339-3p has not yet been fully discovered. Therefore, we investigated the role and regulatory mechanism of miR-339-3p in AP.

TNF receptor-associated factor 3 (TRAF3) is an important signal transducer in intracellular signaling pathways and plays a crucial role in the immune system. Similarly, TRAF3 can also be involved in cell immunity, inflammation, and growth as a target gene in the miRNA regulatory pathway. It has been shown that TRAF3 was a target mRNA of miR-29b-3p and interacted with miR-29b-3p to affect ischemia/reperfusion injury and breast cancer [22,23]. Additionally, TRAF3 was identified to be a target mRNA of miR-455, miR-322, miR-17-92, and miR-188-3p in cerebral ischemic stroke, ischemia/reperfusion injury, and gastric cancer [24,25,26,27]. However, the function of TRAF3 in AP has not been fully clarified.

In this study, we also constructed this model to observe the regulation mechanism of miR-339-3p on inflammatory response and cell necrosis in AP. Finally, we found that miR-399-3p affected cell inflammation and cell apoptosis through targeting TRAF3 via the p38 pathway in caerulein-induced AP. This paper will present a new regulatory pathway for pancreatitis and provide new therapeutic targets for the treatment of pancreatitis.

## 2 Materials and methods

### 2.1 Cell culture and treatment

In this experiment, a rat pancreatic acinar cell line, AR42J, was stimulated by caerulein to construct an AP model. The rat pancreatic acinar cell line AR42J was purchased from the Shanghai Cell Bank of the Chinese Academy of Sciences. The cells were cultured in F12-K medium containing 10% fetal bovine serum and 100 U/mL penicillin and 100 µg/mL streptomycin at 37°C, 5% carbon dioxide, and 95% air and humidity in an incubator. Then, the AR42J cells were treated with 0, 25, 50, and 100 nM caerulein, and after 8 h of culture, the samples were collected and stored at −80°C. In addition, the AR42J cells were treated with 100 nM caerulein, and collected at different times, respectively, at 0, 2, 4, 6, and 8 h and then stored at −80°C for subsequent experiments.

### 2.2 Transfection

The oligomers of miR-339-3p and the fragment of the TRAF3 3′-UTR were synthesized by Sangon Biotech (Shanghai, China). The cells were transfected with the negative control miRNA (NC) and promotion miR-339-3p (miR-339-3p) using Lipofectamine 2000 reagent (Invitrogen) according to the manufacturer’s instructions. For rescue experiments, negative control vector or pcDNA-TRAF3 (TRAF3) and a negative control miRNA (NC) or promotion miR-339-3p (miR-339-3p) were co-transfected into cells using Lipofectamine 2000 reagent (Invitrogen). pcDNATM3.1/CAT is a mammalian expression vector which improves TRAF3 expression. The fragment of TRAF3 was inserted into pcDNATM3.1 with *Xho* I and *Xba* I.

### 2.3 Quantitative real-time PCR

Total RNA was isolated from cells using TRIzol reagent (Invitrogen, Carlsbad, CA, USA). cDNA was reverse transcribed using a High-Capacity cDNA Reverse Transcription Kit (Applied Biosystems, Foster City, CA, USA) for mRNA according to the manufacturer’s instructions. The expression of miR-339-3p was quantified using the TaqMan MicroRNA Expression Assay (Applied Biosystems), according to the manufacturer’s instructions. The fold change in the expression of miR-339-3p and TRAF3 was calculated by the 2^−ΔΔCT^ method with U6 and GAPDH as internal controls. The process of amplification of miR-399-3p was conducted at 95°C for 5 min, then 40 cycles at 95°C for 20 s, 60°C for 25 s, and 72°C for 10 s. The process of amplification of TRAF3 was conducted at 95°C for 10 min, then 40 cycles at 95°C for 20 s, 60°C for 30 s, and 72°C for 10 s. Real-time PCR was performed using SYBR Green (ABI, Foster City, USA). The primers are listed as follows:

miR-339-3p forward: 5′-GGGTGAGCGCCTCGGCGACA-3′; reverse: 5′-CAGTGCGTGTCGTGGAGT-3′

U6 forward: 5′-CTCGCTTCGGCAGCACA-3′; reverse: 5′-AACGCTTCACGAATTTGCGT-3′

TRAF3 forward: 5′-ACTGCAAGAGTCAGGTTCCG-3′; reverse: 5′-CAAGTGTGCACTCAACTCGC-3′

GAPDH forward: 5′-TCCCACTCTTCCACCTTCGA-3′; reverse: 5′-AGTTGGGATAGGGCCTCTCTTG-3′

### 2.4 Western blot

After caerulein treatment, total protein samples were extracted from cells and separated by SDS-polyacrylamide gel electrophoresis. Next, the proteins were transferred onto a polyvinylidene difluoride membrane (Millipore, Bedford, MA, USA). After blocking with 5% non-fat milk, the membrane was incubated with primary antibodies against TRAF3 (anti-rabbit, 1:500; Santa Cruz, Dallas, TX, USA), Bcl-2 (anti-rabbit, 1:500), C-caspase 3 (anti-rabbit, 1:500), Bax (anti-rabbit, 1:500), p-p38 (anti-rabbit, 1:500), p38 (anti-rabbit, 1:500), and GAPDH (anti-mouse, 1:1,000; Santa Cruz) at 4°C overnight. After washing with TBST, the membrane was incubated with a horseradish peroxidase-conjugated secondary antibody (1:1,000 dilution; Santa Cruz Biotechnology Inc.) for 1 h at room temperature. The blots were measured using a Pierce ECL Plus Substrate (Thermo Scientific, USA) according to the manufacturer’s instructions.

### 2.5 Luciferase reporter assay

To explore whether miR-339-3p could directly target TRAF3, a luciferase reporter assay was performed. The fragments containing the wild-type and mutant-type TRAF3 were amplified from 3′-UTR full-length TRAF3 and then were inserted into the luciferase reporter vector psiCHECK-2 (Promega, Madison, WI, USA). Next, the TRAF3-wt and TRAF3-mut were co-transfected with NC and miR-339-3p into the cells using Lipofectamine 2000 reagent (Invitrogen). After transfection for 48 h, firefly and Renilla luciferase activities were analyzed using a Dual-Luciferase Reporter Assay System (Promega, Madison, WI, USA). All transfection experiments were applied at least three times.

### 2.6 Enzyme-linked immunosorbent assay (ELISA)

According to the instructions, the levels of TNF-α, IL-1β, and IL-6 were detected using an Enzyme Immunoassay Kit (Boster, Inc., Wuhan, China). Briefly, when the macrophages reached 70% fusion, the cell supernatant was collected, the antigen was added and kept at 4°C overnight, and then enzyme-labeled secondary antibody (Abcam Inc., Cambridge, MA, USA) was added and incubated at room temperature for 1 h. Finally, the optical density of each sample at 450 nm was measured, and the levels of TNF-α, IL-1β, and IL-6 were calculated according to the regression equation of the standard curve.

### 2.7 RNA immunoprecipitation (RIP) assay

An RIP experiment was performed using a Magna RNA-binding protein immunoprecipitation kit (Millipore, Billerica, MA, USA). The cells were co-transfected with NC, miR-339-3p, or TRAF3 and then incubated with Proteinase K to isolate the immunoprecipitated RNA. After all, the cells were incubated with negative control normal mouse IgG or human anti-Ago2 antibody, RNA was extracted, and the expression of TRAF3 was detected using qRT-PCR.

### 2.8 Cell apoptosis

An Annexin-V-PE/7-AAD double staining apoptosis kit (BD, Franklin Lakes, NJ, USA) was applied to detect cell apoptosis according to the manufacturer’s instructions. Briefly, the cells were resuspended in Annexin V-FITC binding buffer and mixed with Annexin V-FITC and propidium iodide and incubated for 30 min. Then, cell apoptosis rates were analyzed using flow cytometry.

### 2.9 Statistical analysis

All values were expressed as mean ± SD. All data were analyzed using SPSS software and GraphPad Prism 7.0. The statistical significance of differences between groups was determined by Student’s *t*-test. *P* < 0.05 was considered as statistically significant.

## 3 Results

### 3.1 Caerulein induced inflammation and apoptosis in AR42J cells

**Figure 1:**
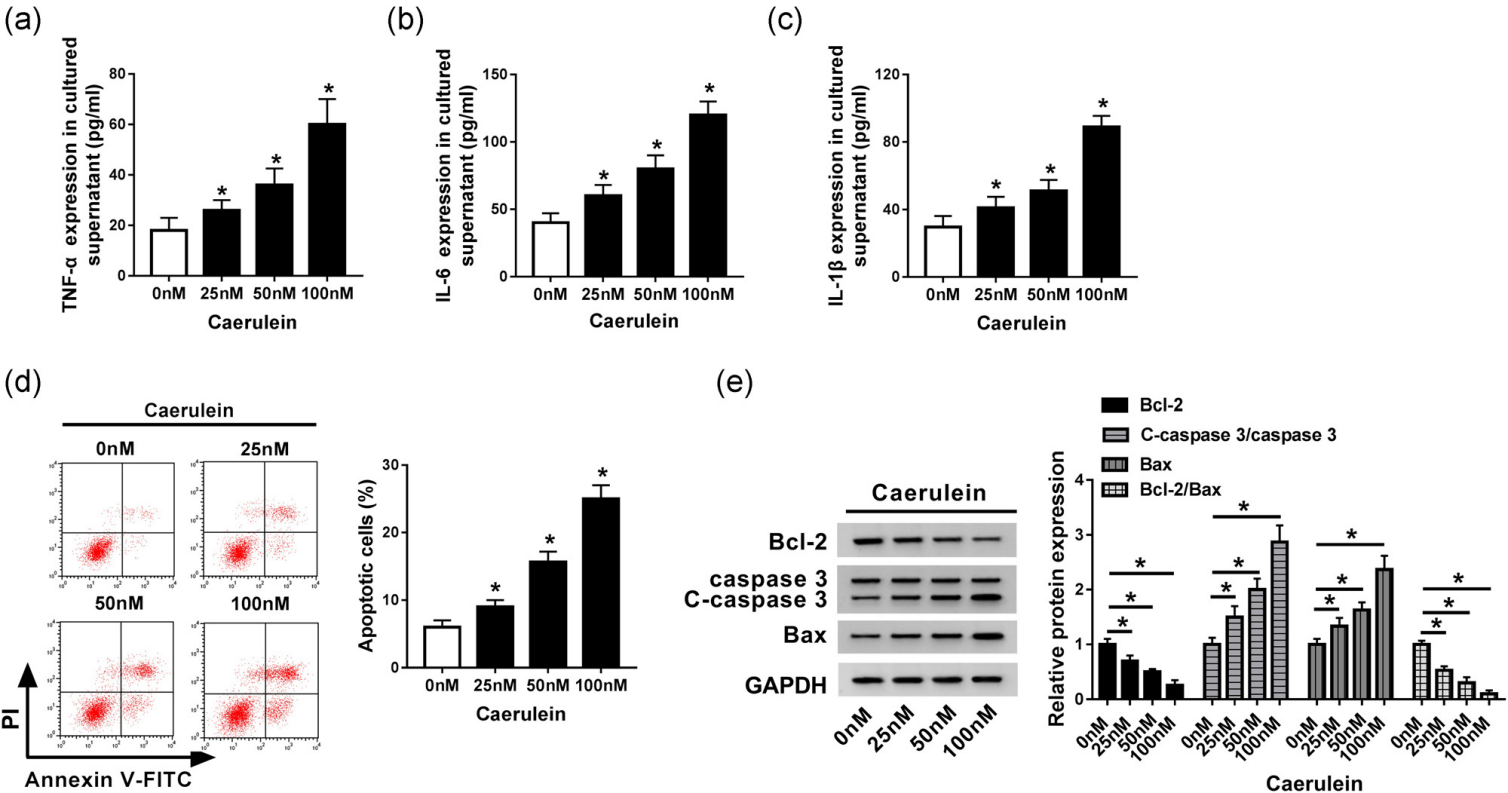
Caerulein induced inflammation and apoptosis in AR42J cells. (a–c) ELISA was used to detect the contents of TNF-α (a), IL-6 (b), and IL-1β (c) in AR42J cells induced by caerulein. (d) Flow cytometry was applied to detect cell apoptosis in AR42J cells induced by caerulein. (e) Western blot was applied to measure the protein expression of Bcl-1, C-caspase 3/caspase 3, Bcl-2/Bax, and Bax in AR42J cells induced by caerulein. Data are represented as mean ± SD of at least triple experiments. *P < 0.05.

First, we determined the cellular inflammatory factor content using ELISA kits. Cell apoptosis and apoptotic factor protein expression were determined using flow cytometry and western blot, respectively. ELISA results displayed that the expression of TNF-α, IL-6, and IL-1β showed similar changes under different concentrations. As the treatment concentration of caerulein increases, their content continued to increase significantly (Figure 1a–c). Meanwhile, flow cytometry analysis showed that cell apoptosis was obviously induced by caerulein (Figure 1d). In addition, the results of western blot showed that the protein expression of Bcl-2 and Bcl-2/Bax was decreased gradually with the increase of the concentration of caerulein, while the protein expression of C-caspase 3/caspase 3 and Bax was enhanced gradually (Figure 1e). The above data indicated that caerulein induced inflammatory response and apoptosis in AR42J cells suggesting that *in vitro* model of AP was characterized by a strong inflammatory response and high cell apoptosis.

### 3.2 miR-339-3p expression was inhibited while TRAF3 expression was increased in AR42J cells induced by caerulein

**Figure 2:**
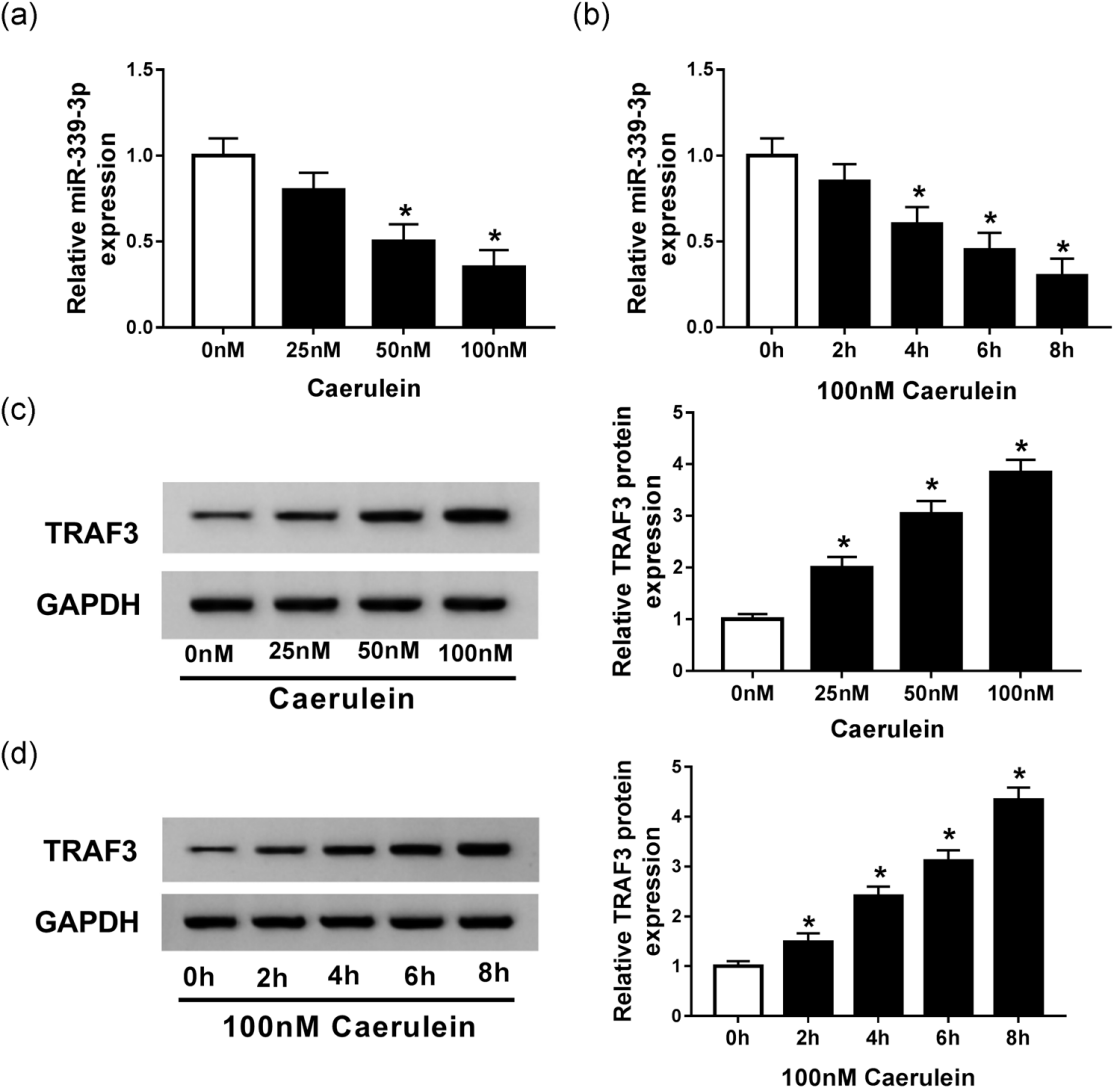
miR-339-3p expression was inhibited while TRAF3 expression was increased in AR42J cells induced by caerulein. (a) qRT-PCR was applied to determine the expression of miR-339-3p under different caerulein concentrations (0, 25, 50, and 100 nM). (b) qRT-PCR was applied to determine the expression of miR-339-3p under 100 nM caerulein concentration at different times (0, 2, 4, 6, and 8 h). (c) Western blot was used to measure the protein expression of TRAF3 under different caerulein concentrations (0, 25, 50, and 100 nM). (d) Western blot was used to measure the protein expression of TRAF3 under 100 nM caerulein concentration at different times (0, 2, 4, 6, and 8 h). Data are represented as mean ± SD of at least triple experiments. *P < 0.05.

Next, we constructed a model of AP by treating AR42J cells with different concentrations of caerulein. As shown in Figure 2a and c, qRT-PCR analysis showed that the expression level of miR-339-3p decreased significantly as caerulein concentration increased. In contrast, western blot analysis showed that the protein expression of TRAF3 was obviously induced with increasing caerulein concentration. In the 100 nM caerulein treatment, the expression of miR-339-3p was significantly lower than that under other concentrations, and the expression of TRAF3 was significantly higher than in other treatments, so we chose 100 nM caerulein treatment for the next experiment. Next, we found that the expression of miR-339-3p was significantly reduced with increasing the processing time, while the expression of TRAF3 was significantly promoted (Figure 2b and d). These results indicate that miR-339-3p and TRAF3 may play an important role in the pathogenesis of AP.

### 3.3 Promotion of miR-339-3p weakened the effect of caerulein on cell inflammation and apoptosis in AR42J cells

**Figure 3:**
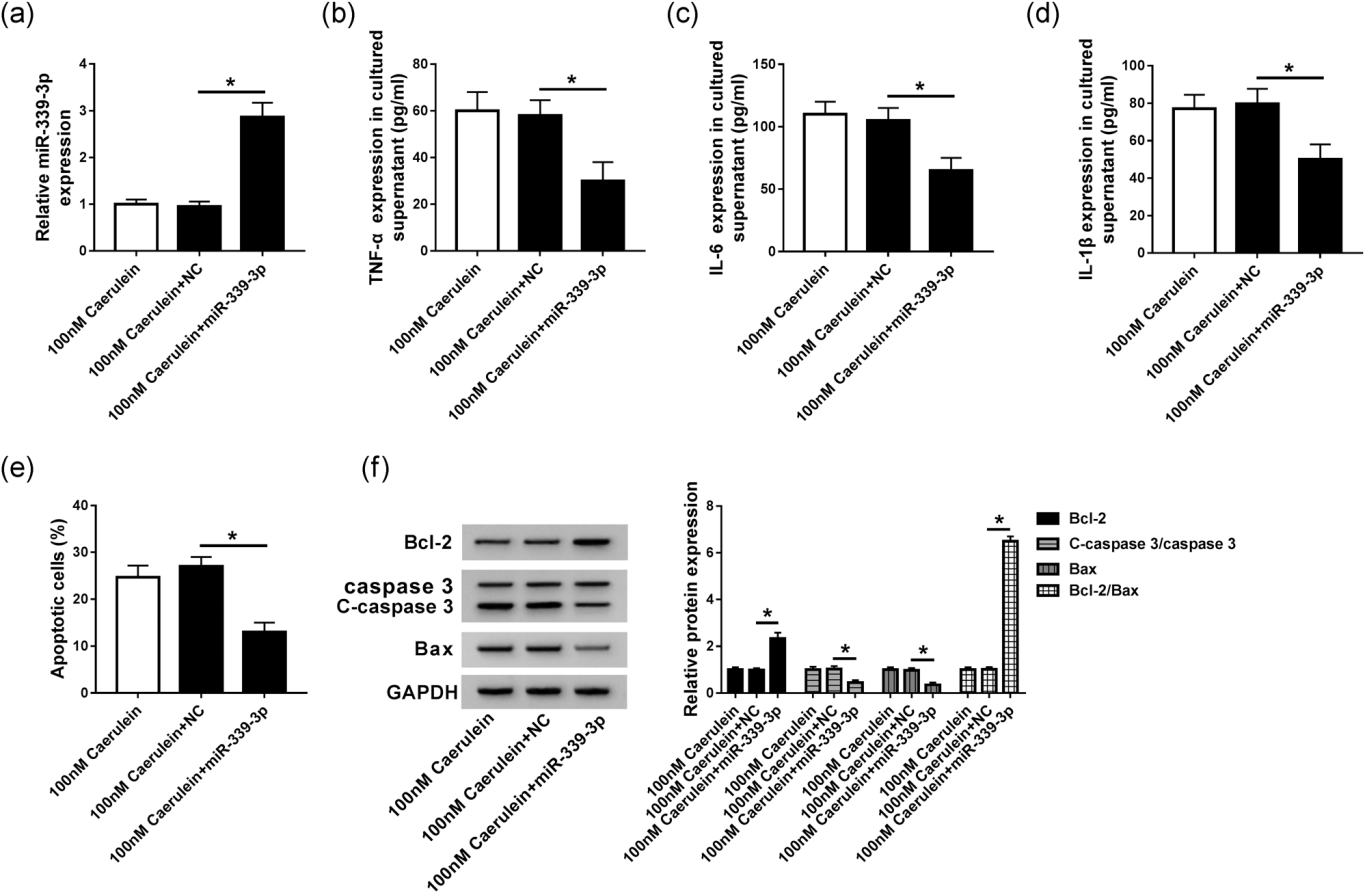
Promotion of miR-339-3p weakened the effect of caerulein on cell inflammation and apoptosis in AR42J cells. (a) The expression of miR-339-3p was detected in 100 nM caerulein, 100 nM caerulein + NC, and 100 nM caerulein + miR-339-3p groups using qRT-PCR. (b–d) ELISA was used to detect the contents of TNF-α (b), IL-6 (c), and IL-1β (d) in 100 nM caerulein, 100 nM caerulein + NC, and 100 nM caerulein + miR-339-3p groups. (e) Flow cytometry was applied to detect cell apoptosis in 100 nM caerulein, 100 nM caerulein + NC, and 100 nM caerulein + miR-339-3p groups. (f) Western blot was applied to measure the protein expression of Bcl-1, C-caspase 3/caspase 3, Bcl-2/Bax, and Bax in 100 nM caerulein, 100 nM caerulein + NC, and 100 nM caerulein + miR-339-3p groups. 100 nM caerulein + NC were used as a negative control. Data are represented as mean ± SD of at least triple experiments. *P < 0.05.

To further explore the function of miR-339-3p in AP, miR-339-3p was transfected into 100 nM caerulein-induced AR42J cells, which increased the expression of miR-339-3p (Figure 3a). We discovered that the levels of TNF-α, IL-6, and IL-1β were lower in the 100 nM caerulein + miR-339-3p group than those in 100 nM caerulein + NC and 100 nM caerulein groups (Figures 3b–d). Additionally, as shown in Figure 3e, we found that cell apoptosis was remarkably inhibited by miR-339-3p transfection in 100 nM caerulein-induced AR42J cells. Moreover, compared with 100 nM caerulein + NC and 100 nM caerulein groups, the protein levels of Bcl-2 and Bcl-2/Bax were significantly improved while the protein levels of C-caspase 3/caspase 3 and Bax were significantly decreased in the 100 nM caerulein + miR-339-3p group (Figure 3f). These results indicated that overexpression of miR-339-3p weakened the effects of caerulein on cell inflammation and apoptosis in AR42J cells, implying that the upregulation of miR-339-3p might inhibit AP.

### 3.4 TRAF3 is a target of miR-339-3p

**Figure 4:**
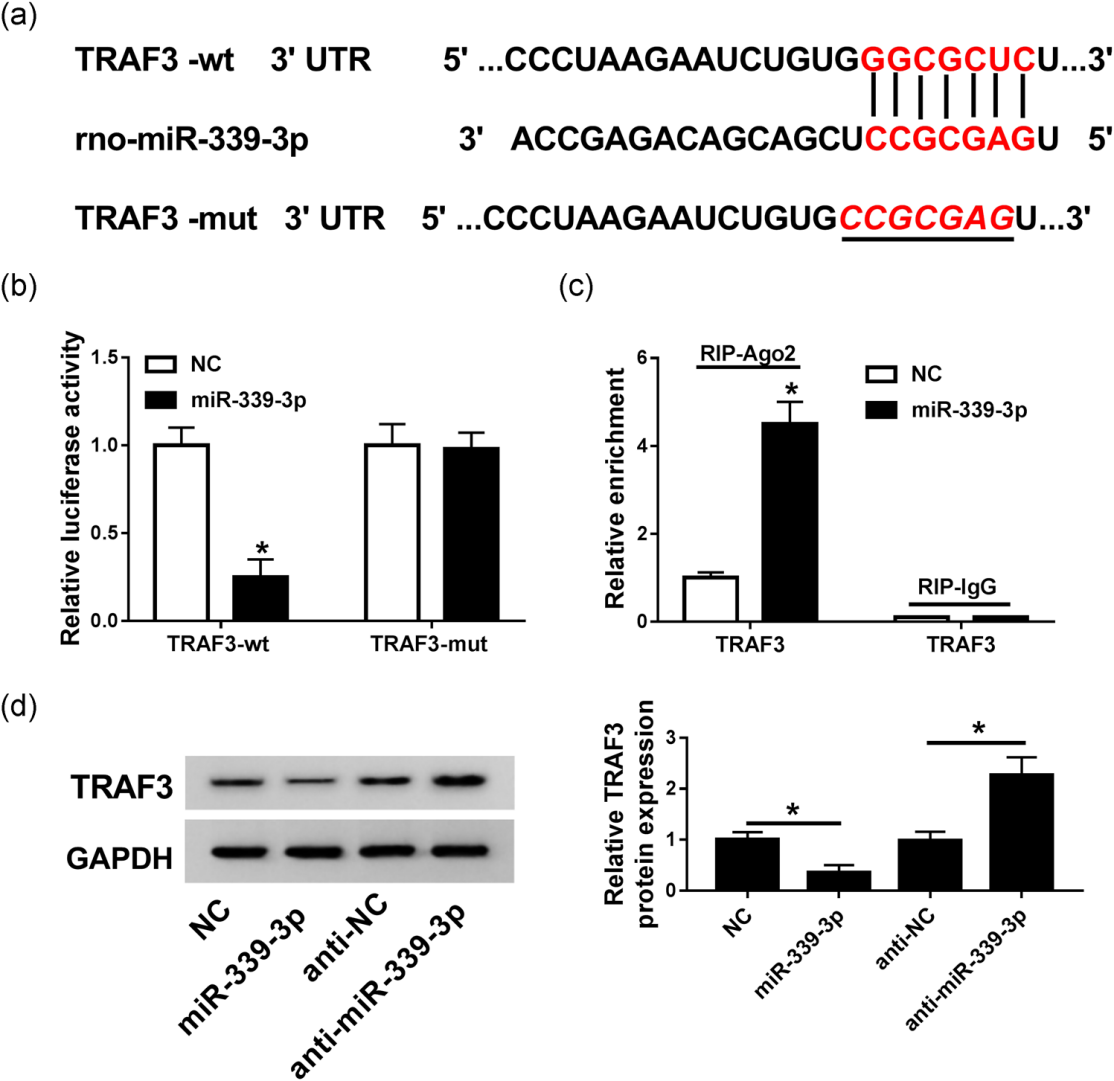
TRAF3 is a target mRNA of miR-339-3p. (a) The binding sites between TRAF3 and miR-339-3p were predicted by TargetScanHuman. (b) Luciferase activities of TRAF3-wt and TRAF3-mut were detected in NC and miR-339-3p groups using the luciferase reporter assay. NC was used as a negative control. (c) The enrichment of TRAF3 was measured in NC and miR-19b-3p groups using the RIP assay. (d) Western blot was used to detect the protein expression of TRAF3 in NC, miR-339-3p, anti-NC, and anti-miR-339-3p groups. NC or anti-NC were used as a negative control. Data are represented as mean ± SD of at least triple experiments. *P < 0.05.

To further explore the regulatory network of miR-339-3p, we successfully found that TRAF3 is a target of miR-339-3p, and miR-339-3p targeted the TRAF3 3′-UTR (Figure 4a). We then used the luciferase reporter assay to analyze this result and constructed luciferase reporters containing the wild-type TRAF3 or mutant sites of TRAF3. As shown in Figure 4b, luciferase activity was significantly reduced when miR-339-3p was bound to TRAF3-wt, whereas the activity was not changed when miR-339-3p was bound to TRAF3-mut (Figure 4b). Otherwise, the RIP analysis showed that the endogenous TRAF3 was particularly enriched in the cells of the miR-339-3p transfected group compared with the NC group (Figure 4c). Furthermore, the protein expression of TRAF3 was measured in the NC group, the miR-339-3p group, the anti-NC group, and the anti-miR-399-3p group using western blot. The results showed that overexpression of miR-399-3p could inhibit the expression of TRAF3, while suppression of miR-399-3p expression could enhance the expression of TRAF3 (Figure 4d). Therefore, these results determined that TRAF3 is a target of miR-399-3p.

### 3.5 Promotion of TRAF3 could reverse the effects of miR-339-3p on cell inflammation and apoptosis in AR42J cells induced by caerulein

**Figure 5:**
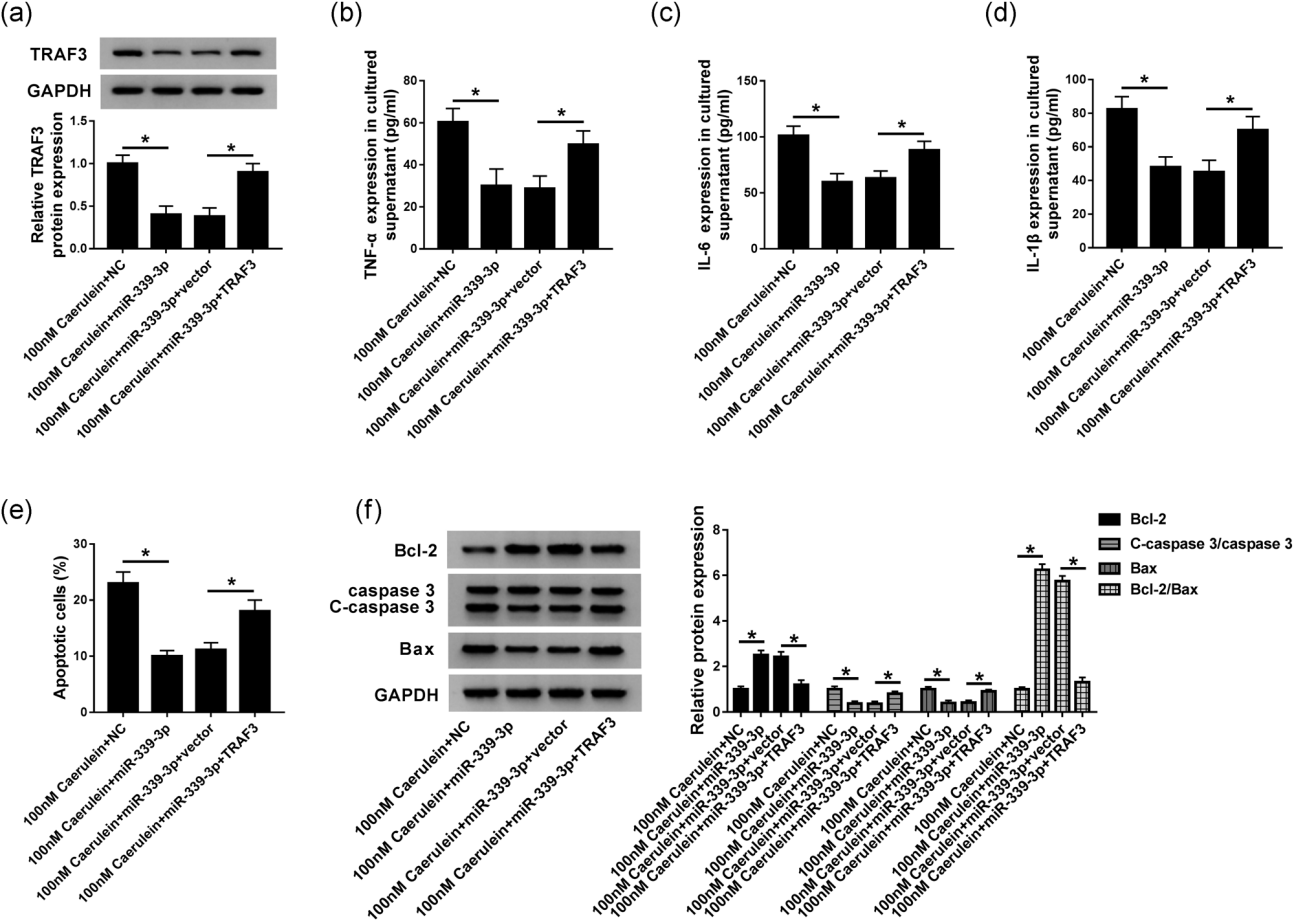
Promotion of TRAF3 could reverse the effects of miR-339-3p on cell inflammation and apoptosis in AR42J cells induced by caerulein. (a) Western blot was used to detect the protein expression of TRAF3 in 100 nM caerulein + miR-339-3p, 100 nM caerulein + NC, 100 nM caerulein + miR-339-3p + vector, and 100 nM caerulein + miR-339-3p + TRAF3 groups. (b–d) ELISA was used to detect the levels of TNF-α (b), IL-6 (c), and IL-1β (d) in 100 nM caerulein + miR-339-3p, 100 nM caerulein + NC, 100 nM caerulein + miR-339-3p + vector, and 100 nM caerulein + miR-339-3p + TRAF3 groups. (e) Flow cytometry was applied to detect cell apoptosis in 100 nM caerulein + miR-339-3p, 100 nM caerulein + NC, 100 nM caerulein + miR-339-3p + vector, and 100 nM caerulein + miR-339-3p + TRAF3 groups. (f) Western blot was applied to measure the protein expression of Bcl-1, C-caspase 3/caspase 3, Bcl-2/Bax, and Bax in 100 nM caerulein + miR-339-3p, 100 nM caerulein + NC, 100 nM caerulein + miR-339-3p + vector, and 100 nM caerulein + miR-339-3p + TRAF3 groups. 100 nM caerulein + NC or 100 nM caerulein + miR-339-3p + vector were used as a negative control. Data are represented as mean ± SD of at least triple experiments. *P < 0.05.

To further explore the role of the regulatory networks of TRAF3 and miR-339-3p in AP, we treated AR42J cells with 100 nM caerulein to construct an AP model, and miR-339-3p, NC, miR-399-3p + vector, and miR-339-3p + TRAF3 were transfected into these cells and divided into 100 nM caerulein + miR-339-3p, 100 nM caerulein + NC, 100 nM caerulein + miR-339-3p + vector, and 100 nM caerulein + miR-339-3p + TRAF3 groups. Western blot analysis indicated that overexpression of miR-339-3p reduced TRAF3 protein expression, but this effect was reversed by the promotion of TRAF3 in AR42J cells induced by caerulein (Figure 5a). Otherwise, we found that the levels of TNF-α, IL-6, and IL-1β were lower in the 100 nM caerulein + miR-339-3p group than in the 100 nM caerulein + NC group. Compared with the 100 nM caerulein + miR-339-3p + TRAF3 group, the levels of TNF-α, IL-6, and IL-1β were decreased significantly in the 100 nM caerulein + miR-339-3p + vector group. These data indicated that the induction of miR-339-3p inhibited inflammation in AR42J cells induced by caerulein, which was impaired by the overexpression of TRAF3 (Figure 5b–d). In addition, high expression of miR-399-3p inhibited cell apoptosis, which was impaired by upregulating TRAF3 (Figure 5e). As shown in Figure 5f, Bcl-2 and Bcl-2/Bax protein levels were induced and C-caspase 3/caspase, Bax, and RIP3 protein levels were reduced by the overexpression of miR-339-3p. However, the transfection of TRAF3 reversed its effect in AR42J cells induced by caerulein. As a result, the promotion of TRAF3 could reverse the effects of miR-399-3p on cell inflammation and apoptosis in caerulein-induced AR42J cells.

### 3.6 miR-399-3p/TRAF3 axis regulated AP induced by caerulein through the p38 pathway

**Figure 6:**
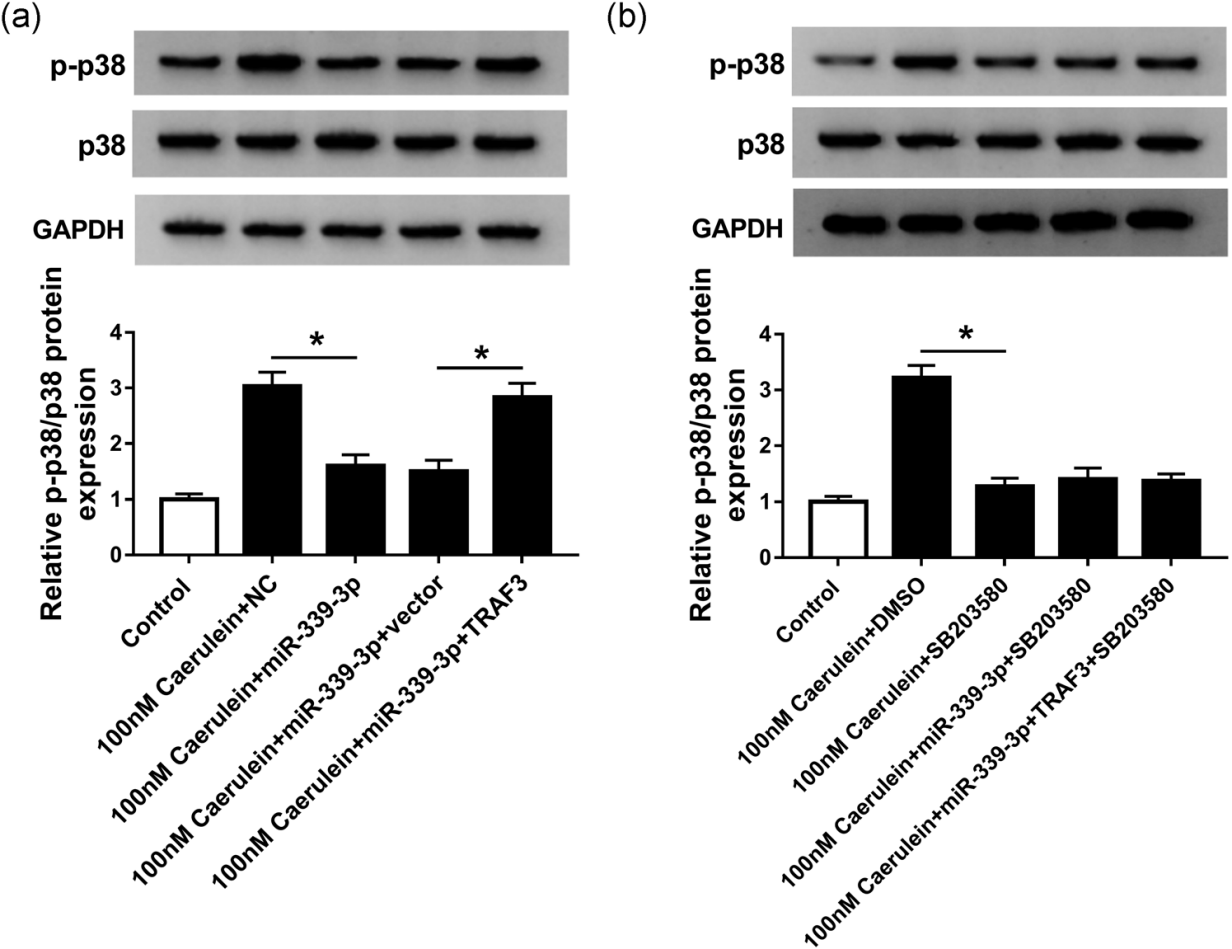
miR-399-3p/TRAF3 axis regulated AP induced by caerulein through the p38 pathway. (a) Western blot was used to detect the protein expression of p-p38 and p38 in 100 nM caerulein + miR-339-3p, 100 nM caerulein + NC, 100 nM caerulein + miR-339-3p + vector, and 100 nM caerulein + miR-339-3p + TRAF3 groups. 100 nM caerulein + NC or 100 nM caerulein + miR-339-3p + vector was used as a negative control. (b) Western blot was used to detect the protein expression of p-p38 and p38 in control, 100 nM caerulein + DMSO, 100 nM caerulein + SB203580, 100 nM caerulein + miR-339-3p + SB203580, and 100 nM caerulein + miR-339-3p + TRAF3 + SB203580 groups. 100 nM caerulein + DMSO or 100 nM caerulein + miR-339-3p + SB203580 were used as a negative control. Data are represented as mean ± SD of at least triple experiments. *P < 0.05.

Western blot results determined that the protein expression of p-p38/p38 was significantly inhibited in the 100 nM caerulein + miR-339-3p group compared with that in the 100 nM caerulein + NC group. However, the protein expression of p-p38/p38 in the 100 nM caerulein + miR-339-3p + vector group was remarkably lower than that in the 100 nM caerulein + miR-339-3p + TRAF3 group (Figure 6a). Besides, SB203580 is an inhibitor of p38 and significantly inhibited the protein expression of p-p38/p38 in AR42J cells treated with 100 nM caerulein (Figure 6b); however, compared with the 100 nM caerulein + miR-339-3p + SB203580 group, the protein expression of p-p38/p38 in the 100 nM caerulein + miR-339-3p + TRAF3 + SB203580 group had no obvious change (Figure 6b). Thus, miR-399-3p suppressed caerulein-induced AP by regulating the p38 pathway via TRAF3.

## 4 Discussion

AP is a common gastrointestinal disease with high morbidity and mortality, and its symptoms are mainly pancreatic necrosis and the inflammatory response. TNF-α, IL-6, and IL-1β are important pro-inflammatory factors. They activate the secretion of macrophages and T-cells in inflammatory signaling and accumulate leukocytes, which are key indicators of inflammatory response [28,29,30]. Bcl-2, C-caspase 3, and Bax proteins are important indicators of cell apoptosis in diseases. Bcl-2 is an important anti-apoptotic protein, and the increase of Bcl-2 can effectively inhibit apoptosis, while C-caspase 3 and Bax protein expressions are significantly elevated in apoptotic cells [31,32,33]. In this study, we constructed an *in vitro* model of AP by inducing rat pancreatic acinar cells with caerulein. We found that, consistent with previous studies, the levels of TNF-α, IL-6, and IL-1β were significantly elevated, and cell apoptosis was promoted in the AP model. The protein expression of C-caspase 3 and Bax was significantly increased, and the protein expression of Bcl-2 was significantly decreased, indicating that AP involves a severe inflammatory reaction and induces apoptosis and necrosis.

More than that, we found that the expression of miR-399-3p was significantly decreased, while the expression of TRAF3 was significantly increased in caerulein-induced *in vitro* model of AP. In this study, the promotion of miR-399-3p significantly inhibited the expression of TNF-α, IL-6, and IL-1β and cell apoptosis and decreased the protein expression of C-caspase 3 and Bax. It is shown that inducing the expression of miR-339-3p can effectively inhibit cellular inflammatory response and apoptosis, and alleviate the symptoms of AP.

The RIP analysis and luciferase reporter assays demonstrated that TRAF3 is a target gene for miR-339-3p, and miR-339-3p transfection significantly inhibited TRAF3 expression. TRAF3 protein encoded by the TRAF3 gene is associated with cell inflammation, immune response, and signal transduction of CD40 [34,35,36]. Interestingly, TRAF3 has been reported to be highly expressed and play an important role in human diseases, such as breast cancer, gastric cancer, and cerebral ischemic stroke [37,38,39]. Moreover, in AP, TRAF3 has been shown to be involved in p38 activation to influence cell inflammation [40,41]. In our study, the promotion of TRAF3 could alleviate the effects of high miR-399-3p expression on pro-inflammatory factors and cell apoptosis in the *in vitro* model of AP. Also, we found that the relative protein expression of caerulein-induced p-p38/p38 was inhibited by overexpression of miR-399-3p, which was impaired by the induction of TRAF3 in the *in vitro* model of AP. Thus, these results indicated that the effect of miR-399-3p on inflammation and apoptosis in the *in vitro* model of AP was mediated by regulating the p38 pathway via TRAF3.

## 5 Conclusion

In this study, we first demonstrated the regulatory network of miR-399-3p and TRAF3. Promotion of miR-399-3p inhibited cell inflammation and cell apoptosis in caerulein-induced *in vitro* model of AP through targeting TRAF3 expression via the p38 pathway, providing a new potential therapeutic target in the treatment of AP.
